# Development and delivery cost of digital health technologies for mental health: Application to the Narrative Experiences Online Intervention

**DOI:** 10.3389/fpsyt.2022.1028156

**Published:** 2022-11-07

**Authors:** Luke Paterson, Stefan Rennick-Egglestone, Sean P. Gavan, Mike Slade, Fiona Ng, Joy Llewellyn-Beardsley, Carmel Bond, Andrew Grundy, Joe Nicholson, Dania Quadri, Sylvia Bailey, Rachel A. Elliott

**Affiliations:** ^1^Manchester Centre for Health Economics, Division of Population Health, Health Services Research and Primary Care, University of Manchester, Manchester, United Kingdom; ^2^School of Health Sciences, Institute of Mental Health, University of Nottingham, Nottingham, United Kingdom; ^3^Health and Community Participation Division, Faculty of Nursing and Health Sciences, Nord University, Namsos, Norway; ^4^Nottingham University Business School, The University of Nottingham, Nottingham, United Kingdom; ^5^School of Health Sciences, University of Manchester, Manchester, United Kingdom; ^6^School of Humanities, The University of Nottingham, Nottingham, United Kingdom; ^7^GKT School of Medical Education, King's College London, London, United Kingdom; ^8^Narrative Experiences Online Intervention (NEON) Lived Experience Advisory Panel, Nottingham, United Kingdom

**Keywords:** narrative, psychosis, mental health, recovery, healthcare costs, digital health intervention, online

## Abstract

**Background:**

The increasing development and use of digital health interventions requires good quality costing information to inform development and commissioning choices about resource allocation decisions. The Narrative Experiences Online (NEON) Intervention is a web-application that delivers recorded mental health recovery narratives to its users. Two randomized controlled trials are testing the NEON Intervention in people with experience of psychosis (NEON) and people experiencing non-psychosis mental health problems (NEON-O).

**Aim:**

This study describes and estimates the cost components and total cost of developing and delivering the NEON Intervention.

**Materials and methods:**

Total costs for the NEON Trial (739 participants) and NEON-O Trial (1,024 participants) were estimated by: identifying resource use categories involved in intervention development and delivery; accurate measurement or estimation of resource use; and a valuation of resource use to generate overall costs, using relevant unit costs. Resource use categories were identified through consultation with literature, costing reporting standards and iterative consultation with health researchers involved in NEON Intervention development and delivery. Sensitivity analysis was used to test assumptions made.

**Results:**

The total cost of developing the NEON Intervention was £182,851. The largest cost components were software development (27%); Lived Experience Advisory Panel workshops (23%); coding the narratives (9%); and researchers' time to source narratives (9%). The total cost of NEON Intervention delivery during the NEON Trial was £118,663 (£349 per NEON Intervention user). In the NEON-O Trial, the total delivery cost of the NEON Intervention was £123,444 (£241 per NEON Intervention user). The largest cost components include updating the narrative collection (50%); advertising (19%); administration (14%); and software maintenance (11%). Uncertainty in the cost of administration had the largest effect on delivery cost estimates.

**Conclusion:**

Our work shows that developing and delivering a digital health intervention requires expertise and time commitment from a range of personnel. Teams developing digital narrative interventions need to allocate substantial resources to curating narrative collections.

**Implications for practice:**

This study identifies the development and delivery resource use categories of a digital health intervention to promote the consistent reporting of costs and informs future decision-making about the costs of delivering the NEON Intervention at scale.

**Trial registration:**

NEON Trial: ISRCTN11152837, registered 13 August 2018, http://www.isrctn.com/ISRCTN11152837. NEON-O Trial: ISRCTN63197153, registered 9 January 2020, http://www.isrctn.com/ISRCTN63197153.

## Introduction

In recent years, there has been a rapid increase in digital health technologies (DHTs) to deliver mental health interventions remotely, either to replace or supplement face-to-face healthcare, with that increase accelerating during the COVID-19 pandemic (1, 2).

Digital health technologies range from predefined functions such as remote diagnosis or disease monitoring, to more complex functions around supporting behavior change through interactive and personalized interactions. A sub-type of DHTs are digital health interventions (DHIs), which provide users with remote access to treatment through text messaging services, smartphone apps, and web-based resources. Recent reviews of the growing number of trials have shown promising but varied effectiveness for DHIs in a wide range of mental health conditions, including but not limited to depression (3, 4), eating disorders (5), post-traumatic stress disorder (6), and schizophrenia (7).

Investment in mental health DHIs is increasing, partly to improve access to care in overstretched health systems and partly due to emerging patient and user preference for DHI delivery of mental health care (4). This means that decisions are being made about effectiveness and affordability of individual DHIs. Economic evaluations inform healthcare resource allocation decisions and treatment recommendations by comparing the costs and health benefits of alternative ways to treat patients (8). Demonstrating cost-effectiveness is an important factor for delivering DHIs into healthcare systems across Europe (9). Economic evaluations of mental health DHIs have been conducted for a range of conditions such as anxiety (10, 11), depression (10–12), eating disorders (10, 13), and substance abuse disorders (10, 14). The quality of economic evaluations has been suggested to be very variable including heterogeneous reporting of costs, outcomes and comparators (15). Such inconsistencies may be a barrier to the adoption of effective DHIs for mental health within healthcare systems or may lead policymakers to invest in services that are not cost-effective (16–18).

There is a lack of standardization in how costs are reported and described for economic evaluations of DHIs (11, 15). The National Institute of Health and Care Excellence's (NICE) evidence standards framework for DHTs recommends reporting all costs associated with the intervention and costs relevant to a health and social care decision maker (19). The framework focuses on delivery (implementation) costs, as these are relevant to health and social care providers looking to commission a service, and includes initial investment costs such as training, as well as operation, and maintenance costs (19). Apart from the initial fixed set-up costs, including infrastructure and training costs, evolution of the DHI over time (interface design, software updates, and content updates) requires more flexible data collection tools that can keep pace with these changes. For example, the marginal cost is the additional cost incurred when one more person uses the product. DHIs tend to have a low marginal cost (one more user accessing an app tends to a zero marginal cost) up to a certain threshold, beyond which there is a large increase due to the need to re-engineer components of technology (such as centralized servers maintaining user account records and delivering web-based content) to provide additional capacity. Other challenges for DHI costing include having to respond to rapidly changing prices (such as exchange rate fluctuations) and short life-cycles of technology, short depreciation periods, and attribution of cost from a shared resource such as a wireless network (20).

Development costs are not typically considered in economic evaluations as, by definition, they have already been incurred prior to delivery. Historically, these development costs are sunk costs, and may include cross-subsidization of product development where there is a failure to reach product launch. Development costs can be included in a market price, which will fall as the market share, or the number of users increases. A recent systematic review reported development costs were only reported in four out of 24 economic evaluations of internet-based interventions for anxiety and depression (11). There are no guidelines for estimating the development cost of DHIs, resulting in non-standardized approaches and a lack of comparability across studies. However, development costs are relevant to publicly funded research groups, research funders, university enterprise offices and private companies and by informing decisions to develop DHIs or scale an existing technology. DHI development incurs significant research and development costs prior to launch, and the resources consumed during development can be very different from pharmaceuticals and physical medical devices. DHIs have other unique characteristics that can affect development processes and thus costs, including faster evolution, active and more dynamic user input (21). DHI development costs are usually incurred for content and software design, website and graphic design, digital platform development and regulatory approval processes. A recent study focusing on the development of a mental health DHI proposed a costing framework for development costs and we have applied this framework to our development costs methods (22).

The aim of this study is to describe, estimate, and present the associated cost components and total cost of developing and delivering the Narrative Experiences Online (NEON) Intervention, using current recommendations for costing DHIs (11, 19, 21, 22).

### Digital health intervention under investigation: NEON Intervention

The NEON Intervention is a web-based application that delivers recorded recovery narratives to its users. A systematic review (23) and qualitative validation study (24) defined mental health recovery narratives as first-person lived experience accounts of recovery from mental health problems, which include elements of adversity or struggle and of self-defined strengths, successes or survival. The impact of recovery narratives was then investigated in a systematic review (25), qualitative interviews (26), and experimental studies (27, 28). Approaches to curation of recorded recovery narrative collections were developed through systematic review (29), stakeholder consultation (30) and best practice guidelines development (31). These studies, together with related work on post-traumatic growth (32, 33), non-service user perspectives (34), institutional injustice (35), and clinician perspectives on use of narratives in practice (36), provided the theory base.

The NEON Intervention was then developed (37). The NEON Intervention allows recipients to engage with recovery narratives by watching, reading, or listening to narrator-led stories on the website. Access to these narratives is provided through different avenues: a hybrid recommender system using collaborative and content-based filtering to recommend appropriate stories; self-selected stories from the entire collection of narratives (referred to as the NEON Collection); randomly selected stories; recommendations sent to users in emails, to serve as a mechanism for engaging people with the intervention; and re-requested narratives that have been previously seen. The recommender system uses feedback data from stories received by participants, characteristics of the participants, and characteristics of each recorded recovery narrative assessed using the Inventory of Characteristics of Recovery Stories (INCRESE) measure (38), to match participants to narratives intended to be of benefit. The website was engineered to work on personal computers, mobile devices, or communal computers such as in a public library to enable participation by people experiencing digital exclusion (39).

An economic evaluation is being conducted as part of two definitive randomized controlled trials (RCTs) to evaluate the cost-effectiveness of offering the NEON Intervention to individuals with experience of psychosis (NEON Trial, *n* = 739) and people experiencing non-psychosis mental health problems (NEON-O Trial, *n* = 1,024) (40, 41).

This study reports the estimation of development and delivery costs for the NEON Intervention, which is used in both trials.

## Materials and methods

This study was conducted as part of the Narrative Experiences Online (NEON) Programme (researchintorecovery.com/neon), which is investigating whether receiving recorded mental health recovery narratives improves the quality of life in people who experience mental health issues.

### Design of costing study

Estimated total cost and individual cost components for the development and delivery of the NEON Intervention are reported. The development costs included those incurred from the perspective of the research body who funded the development and testing of the NEON Intervention. The delivery costs included those incurred from the perspective of the health and social care provider (National Health Service, NHS England) (19). The length of time over which delivery costs were collected was from the beginning of delivery (9th March 2020) to the end of the trial periods (13th May 2022 for NEON Trial, 23rd June 2022 for NEON-O Trial). The NEON Intervention was identical for both NEON and NEON-O trials, since both trials provided participants with access to the same narrative collection. A separate cost of delivery of the NEON Intervention is presented for both trials. Downstream costs (those incurred as a result of using the intervention from the perspective of the NHS) will be reported in a subsequent full economic evaluation.

The development costs begin from the start of the current research program funding, comprising software and intervention development, through feasibility testing and up to the starting point of the definitive trial. Early conceptual development work, and research-related tasks including the randomized controlled trials which contribute to a large part of the program grant, are excluded in the development cost estimates. The duration of the development of the NEON Intervention was considered over the period January 2017 to January 2021.

The total cost for the development stage of the NEON Intervention was constructed with reference to a development costing checklist for a digital program for training community health workers to deliver treatment for depression (22). The costing strategy consisted of: identifying resource use categories involved in the development and delivery of the intervention; accurate measurement or estimation of resource use; and a valuation of resource use to generate overall costs (42). Identifying resource use categories was carried out through consultation with published literature relevant to DHIs, costing reporting standards and expert consultation with team members involved in intervention development and delivery.

The product of resource use and unit costs generated total cost estimates, and this is referred to as the base-case analysis. In this study, the resource use data obtained for development and delivery was obtained for the whole user cohort (top-down costs), rather than data relevant to a specific user (bottom-up costs). Therefore, the approach taken was to generate total development and delivery costs and then apportion to individual users as a “mean cost per user.” The number of users was defined as the number of people randomized to the intervention group (370 in the NEON Trial and 512 in the NEON-O Trial).

In the base-case analysis, costs for human resources were obtained by multiplying the personnel's midpoint hourly salary plus on-costs (pension contributions and payroll taxes) by the proportion of hours spent on the task. For external experts and consultants their costs were recorded as invoices to the NEON study. In some cases where records were not kept, the proportion of hours spent on a task was estimated through expert consultation to produce the maximum and minimum plausible duration; with the midpoint (average) selected during the base-case analysis. For information technologies used in the development and delivery of the NEON Intervention, financial records were used to calculate the cost of the components purchased. In one case, an estimate was derived from the NEON budget proposal.

Deriving estimates of certain cost components required some assumptions to be made with uncertainty further associated with the true values of several components. To assess the impact of our assumptions and parameter uncertainty, all input parameters were adjusted to their extreme values individually in a one-way sensitivity analysis. Tornado diagrams (43) were used to illustrate which input parameters had the most impact on cost estimates. Structural uncertainty resulting from the assumptions made were examined through scenario analysis.

This costing study was developed and reported in accordance to standard validation and reporting criteria (44). A team member not involved in the analysis used the Consolidated Health Economic Evaluation Reporting Standards (CHEERS) 2022 checklist to ensure that relevant items were reported completely (see Appendix 1). Face validity of cost categories and costing methods was ascertained through continuous feedback from clinical and patient experts. All costs are presented in UK Sterling (£) for the costing period 2020–2021.

### Costing the NEON Intervention

We identified several resource use categories in intervention development and delivery, summarized in Table 1. This section provides detail on how resource use and unit costs were obtained for each category.

**Table 1:** Summary of NEON Intervention development and delivery resource use categories.

**Development resource use categories**
Curation of the NEON Collection: • Staff time to source recovery narrative • Staff time to secure ethical approval • Collection organizers • Lived Experience Advisory Panel workshops • Collection Steering Group meetings (including preparation for meetings) • Training researchers • Coding narratives
Web application development: • Database specification • Software development • Recommender system and integrating into the codebase • Graphic design • Interaction design
Communication: • Task meetings • Advisory Board meetings • Design Group meetings
Intervention: • Intervention testing • Feasibility study
**Delivery resource use categories**
Web hosting
Personnel training
Periodic updates to the narrative collection
Web-application maintenance
Administrative support
Advertisement
Intervention engagement
Safeguarding

#### NEON Intervention development resource use

Four components that incurred resources were needed to develop the NEON Intervention:

- building a collection of recovery narratives (the NEON Collection) that would be used in the intervention (consisting of sourcing narratives, securing ethical approval to use those narratives, liaising with collection organizers to source narratives from existing collections, Lived Experience Advisory Panel (LEAP) workshops to develop recommendations on (1) the ethical issues around narrative curation and (2) the initial curation procedures, Collection Steering Group (CSG) meetings to make decisions on (1) the inclusion of individual narratives and (2) refinement of the curation procedures, training researchers to use the INCRESE tool, coding narratives using INCRESE);
- developing the web-application as a platform to deliver the NEON Intervention [consisting of reporting a database specification to support the development of the web-application, developing source code for the web-application (software development), conceptualizing and developing a recommender system that matches users with the most appropriate narratives, integrating the recommender system into the web-application codebase, and designing the intervention to ensure it was appealing to both operate aesthetically (graphic design) and practically (interaction design)];
- additional communications necessary to develop the intervention [consisting of NEON study team task meetings for general discussions, International Advisory Board (IAB) meetings to advise on safety and ethical concerns, and Intervention Development Group (IDG) meetings to provide (feedback on the intervention)];
- testing the intervention (consisting of testing the intervention's performance on the web-application including the collection of outcome data, and a feasibility study evaluating a prototype of the intervention in a small sample of mental health service users (baseline: *n* = 25; follow-up: *n* = 22) with experience of mental health problems);

Resource use during the development of the NEON Intervention was measured through examinations of records and/or derived estimates with assistance from expert consultation. Tables 2–5 summarize resource use and unit cost categories and sources for the four components of NEON intervention development.

**Table 2:** Development resource use and unit cost input parameters: NEON Collection curation.

**Resource item**	**Staff members and details**	**Quantity consumed (range)**	**Method**	Cost per unit*(range)	**Source**
Staff time to source recovery	APM3	12.5 days (10–15 days)	Expert estimation	£23.90 per hr (£19.92–£28.72)	[The University of Nottingham's Aug 2021 Clinical Salary Scales]
narrative	RA	65 days (50–85 days)		£28.80 per hr (£23.18–£34.47)	
Staff time to secure ethical	CI	2.5 days (2–3 days)		£69.91 per hr (£46.49–£102.26)	[Glassdoor March 2022]
approval	SRF	12.5 days (10–15 days)		£41.34 per hr (£34.47–£49.54)	[The University of Nottingham's Aug 2021 Clinical Salary Scales]
Collection organizers	N/A	N/A	Direct observation	£2.79 per collection organizer source narrative	[Invoice to the NEON study]
LEAP workshop	APM3	23.4 hr (13.7–33.2 hrs)	Direct observation with assumption	£23.90 per hr (£19.92–£28.72)	[The University of Nottingham's Aug 2021 Clinical Salary Scales]
	RA			£28.80 per hr (£23.18–£34.47)	
	SRF			£41.34 per hr (£34.47–£49.54)	
	CI			£69.91 per hr (£46.49–£102.26)	[Glassdoor March 2022]
	10 LEAP members			£20 per hr	[Internal communication with NEON study team]
	Travel/venue	13 workshops	Direct observation	£128 per LEAP member meeting	[Invoice to the NEON study]
Collection Steering Group	RA	14 hr	Direct observation	£28.80 per hr (£23.18–£34.47)	[The University of Nottingham's Aug 2021 Clinical Salary Scales]
Meetings	SRF			£41.34 per hr (£34.47–£49.54)	
	4 LEAP members			£20 per hr	[Internal communication with NEON study team]
	APM3; preparation for meetings	7.25 hr		£23.90 per hr (£19.92–£28.72)	[The University of Nottingham's Aug 2021 Clinical Salary Scales]
Training	APM3	5 hr (3.75–6.25 hr)	Expert estimation	£23.90 per hr (£19.92–£28.72)	[The University of Nottingham's Aug
researchers	RA	5 hr (3.75–6.25 hr)		£28.80 per hr (£23.18–£34.47)	2021 Clinical Salary Scales]
	SRF	5 hr (3.75–6.25 hr)		£41.34 per hr (£34.47–£49.54)	
Staff time to code the narratives	Researchers	1,092 hr (819–1,638 hr)	Direct observation with assumption	£20 per hr	[Internal communication with NEON study team]

APM3, Administrative, Professional and Managerial level 3 (APM3); CI, Clinical Investigator; hr, hour; LEAP, Lived Experience Advisory Panel; RA, Research Associate; SRF, Senior Research Fellow.

As per the University of Nottingham's “Normal Full-time Working Week,” it was assumed that the number of hours worked per week was 36.25 h for all professionals. Given there were 215 working days in 2021 (including 30 days annual leave, 8 public holidays, and 7 university holidays/closures), the number of hours worked per year was assumed to be 1,559 h.

^*^The unit costs used in the base case model contain direct salary plus on-costs [employer's national insurance contributions, employer's Universities Superannuation Scheme (USS) pension contribution, and the apprenticeship levy].

**Table 3:** Development resource use and unit cost input parameters: Web-application development.

**Resource item**	**Staff members and details**	**Quantity consumed**	**Method**	Cost per unit*	**Source**
Database specification	SRF	4.5 days (4–5 days)	Expert estimation	£41.34 per hr (£34.47–£49.54)	[The University of Nottingham's Aug 2021 Clinical Salary Scales]
Software development	N/A	N/A	Direct observation	100% of invoice	[Invoice to the NEON study]
Recommender system development	Work package 3.1	N/A	Direct observation with assumption	30% of budget	[NEON study budget proposal]
	SRF; integrating the algorithm into site	4 days (3–5 days)	Expert estimation	£41.34 per hr (£34.47–£49.54)	[The University of Nottingham's Aug 2021 Clinical Salary Scales]
Graphic design	N/A	N/A	Direct observation	100% of invoice	[Invoices to the NEON study]
Interaction design	SRF	4 days (3–5 days)	Expert estimation	£41.34 per hr (£34.47–£49.54)	[The University of Nottingham's Aug 2021 Clinical Salary Scales]

hr, hour; SRF, Senior Research Fellow.

As per the University of Nottingham's “Normal Full-time Working Week,” it was assumed that the number of hours worked per week was 36.25 h for all professionals. Given there were 215 working days in 2021 (including 30 days annual leave, 8 public holidays, and 7 university holidays/closures), the number of hours worked per year was assumed to be 1,559 h.

^*^The unit costs used in the base case model contain direct salary plus on-costs [employer's national insurance contributions, employer's Universities Superannuation Scheme (USS) pension contribution, and the apprenticeship levy].

**Table 4:** Development resource use and unit cost input parameters: Additional communication.

**Resource item**	**Staff members and details**	**Quantity consumed**	**Method**	Cost per unit*(range)	**Source**
Task meetings	CI	56.8 hrs (13.76–99.8 hrs)	Expert estimation	£69.91 per hr (£46.49–£102.26)	[Glassdoor March 2022]
	RA			£28.80 per hr (£23.18–£34.47)	[The University of Nottingham's Aug 2021 Clinical Salary Scales]
	SRF			£41.34 per hr (£34.47–£49.54)	
	APM3			£23.90 per hr (£19.92- £28.72)	
International	CI	1 hr (0.75–1.25 hrs)	Expert estimation	£69.91 per hr (£46.49–£102.26)	Glassdoor March 2022]
Advisory Board	4 Profs			£69.91 per hr (£46.49–£102.26)	
	SRF			£41.34 per hr (£34.47–£49.54)	[The University of Nottingham's Aug 2021 Clinical Salary Scales]
	2 Profs; further consultations	4 hrs (3–5 hrs)		£69.91 per hr (£46.49–£102.26)	[Glassdoor March 2022]
Intervention Design Group meetings	SRF	1.5 hrs	Direct observation	£41.34 per hr (£34.47–£49.54)	[The University of Nottingham's Aug 2021 Clinical Salary Scales]
	2 LEAP members			£20 per hr	[Internal communication with NEON study team]

APM3, Administrative, Professional and Managerial level 3 (APM3); CI, Clinical Investigator; hr, hour; LEAP, Lived Experience Advisory Panel; Profs, Professors; RA, Research Associate; SRF, Senior Research Fellow.

As per the University of Nottingham's “Normal Full-time Working Week,” it was assumed that the number of hours worked per week was 36.25 h for all professionals. Given there were 215 working days in 2021 (including 30 days annual leave, 8 public holidays, and 7 university holidays/closures), the number of hours worked per year was assumed to be 1,559 h.

^*^The unit costs used in the base case model contain direct salary plus on-costs [employer's national insurance contributions, employer's Universities Superannuation Scheme (USS) pension contribution, and the apprenticeship levy].

**Table 5:** Development resource use and unit cost input parameters: Testing the intervention.

**Resource item**	**Staff members and details**	**Quantity consumed**	**Method**	Cost per unit*	**Source**
Intervention testing	RA	8.5 days (7–10 days)	Expert estimation	£28.80 per hr (£23.18–£34.47)	[The University of Nottingham's Aug 2021 Clinical Salary Scales]
	SRF	13.5 days (12–15 days)		£41.34 per hr (£34.47–£49.54)	
	CI	20 hrs (15–25 hrs)		£69.91 per hr (£46.49–£102.26)	
	STAT	2.5 days (2–3 days)		£50.74 per hr (£40.64–£60.46)	Queen Mary University of London Aug 2021 Salary Scales
Feasibility study
Baseline	RA	3 hrs (2.25–3.75 hrs)	Expert estimation	£28.80 per hr (£23.18–£34.47)	[The University of Nottingham's Aug 2021 Clinical Salary Scales]
	25 participants	3 hrs (2.25–3.75 hrs)		£20 per hr	[Internal communication with NEON study team]
	Transcript	25 transcripts	Direct observation	£72.13 per transcript	[Invoice to the NEON study]
	Travel	25 participants	Expert estimation	£5.15 per participant	[Internal communication with NEON study team]
Follow-up	RA	3hrs (2.25–3.75 hrs)	Expert estimation	£28.80 per hr (£23.18–£34.47)	[The University of Nottingham's Aug 2021 Clinical Salary Scales]
	22 participants	3hrs (2.25–3.75 hrs)		£20 per hr	[Internal communication with NEON study team]
	Transcript	22 transcripts	Direct observation	£41.22 per transcript	[Invoice to the NEON study]
	Travel	22 participants	Expert estimation	£5.15 per participant	[Internal communication with NEON study team]
Analysis	RA	30 hrs (22.5–37.5 hrs)	Expert estimation	£28.80 per hr (£23.18–£34.47)	[The University of Nottingham's Aug 2021 Clinical Salary Scales]
	SRF	10 hrs (7.5–12.5 hrs)		£41.34 per hr (£34.47–£49.54)	

CI, Clinical Investigator; hr, hour; RA, Research Associate; SRF, Senior Research Fellow; STAT, Senior Statistician.

As per the University of Nottingham's “Normal Full-time Working Week,” it was assumed that the number of hours worked per week was 36.25 h for all professionals. Given there were 215 working days in 2021 (including 30 days annual leave, 8 public holidays, and 7 university holidays/closures), the number of hours worked per year was assumed to be 1,559 h.

^*^The unit costs used in the base case model contain direct salary plus on-costs [employer's national insurance contributions, employer's Universities Superannuation Scheme (USS) pension contribution, and the apprenticeship levy].

An essential component of the development of the NEON Intervention was building the NEON Collection (37). The recovery stories used were sourced based on the objective of maximizing the diversity of the types of stories within the collection based on different diversity domains (such as narrator ethnicity, sexuality, gender identity, neurodiversity, etc.). There were two routes in which narratives were sourced for the collection: individual donations (~7% of the collection) and existing collections (~93% of the collection). Both routes required work to secure permission to use the narratives within the NEON Collection. For donated narratives, this involved liaising directly with the narrator to secure permission for the re-use of the story. For narratives sourced from existing collections, the curator either had prior permission for the narratives to be redistributed or they approached the narrators for whom they did not already have the appropriate permission for the re-use of the narratives. The eligibility for a narrative to be adopted within the collection was assessed based on the inclusion/exclusion criteria identified through NEON task meetings and Lived Experience Advisory Panel (LEAP) workshops with any ethical uncertainties resolved during Collection Steering Group (CSG) meetings.

The NEON study LEAP workshops were chaired 13 times with attendance from 10 LEAP members, a LEAP meeting chair, and members of the NEON team. It was estimated that 60% of these workshops were necessary for the development of NEON as opposed to other research-related activities by examining the workshop agendas. The LEAP members had personal experiences of mental health problems and advised on the ethical principles of curating narratives, categorizing narrative content warnings, general issues raised by the research team, and the types of narratives to be included/excluded from the NEON Collection (28). As with all in-person meetings/workshops run by the NEON study all participants were paid for their attendance. Additionally, before the COVID-19 pandemic, travel expenses and hospitality (including venues and refreshments) were covered as expenses and recorded through invoices.

A CSG had the authority to make all final ethical decisions regarding the approval of narratives into the collection where the research team expressed uncertainty surrounding whether all inclusion criteria were met and/or whether an exclusion criterion was met. Moreover, the steering group could make recommended updates to the inclusion and exclusion criteria (see researchintorecovery.com/research/neon/neoncollection). This group was comprised of four LEAP members, a senior research fellow, and a research assistant. In total, there were eight meetings lasting 2 h per session with preparation for the meetings completed by an administrator; referred to as an Administrator, Professional, Managerial level 3 (APM3) within the host university. All the CSG meetings were necessary for the development of the NEON Intervention.

Once permission to use the stories were granted, the narratives were characterized using a standardized 77-item INCRESE tool (38). Researchers were trained to rate narratives to identify latent characteristics (e.g., the stage of recovery, genre) and manifest characteristics (e.g., narrator gender and content warnings). Each narrative was double rated for the content warning section of INCRESE. Training to code the recovery narratives using INCRESE was conducted as a part of a 10-day pilot study to test the validity of the tool, in which 100 narratives were rated. The cost of training researchers to use the INCRESE tool only accounts for the trainers' costs with the trainees' costs captured within the total coding time estimate.

For the process of coding the narratives, the researchers recorded the time taken to read, watch, or listen to the narratives. Although this provides an accurate estimate of the time taken to code the narratives, it does not contain a record of the length of breaks the researchers used during the process. Apart from rest periods being a standard part of a working day, a selection of the recovery stories may have caused an emotional or distressing impact on the researchers. Therefore, including breaks may more closely reflect the practical reality of coding the narratives (37). It was assumed that for every 6 h spent coding the coder had a 2-h break. After the recovery stories had been rated with INCRESE, they were added to the NEON Collection.

The NEON Intervention was delivered through a web-application to its users. A database specification document was required to support database implementation and source code development. Fundamental to the NEON Intervention is a recommender system that matches users with narratives intended to be of benefit. The researchers' time to conceptualize and develop the recommender system was challenging to estimate without records. The proposed budget line from the NEON trials was used as an informed approximation of the cost involved in developing the recommender system. Following this, the recommender system was integrated into the web-application codebase. As both developing and integrating the recommender system into the web-application are related tasks, they were combined into one cost.

An important component of developing the NEON Intervention was ensuring the web-application was appealing to use. Graphic designers made improvements to the aesthetical appeal of the web-application interfaces following feedback during the feasibility study and from LEAP members. The costs of keeping users engaged in the intervention (e.g., gamification, testimonials from other users, etc.) are spread between both graphic design and software development costs. Interaction design was also important to ensure the web-application's functions operated as intended (i.e., navigations).

Throughout the NEON study, task meetings were used to communicate and raise general discussions surrounding the interventions development. An assumption was made, through inspection of meeting agendas, that 33% of task meetings were necessary for the purpose of developing the intervention (i.e., excluding research costs). A total of 86 meetings were held with recorded attendance from members of the NEON team.

The international advisory board was used to provide expert consultation on safety strategies for the development of the NEON Collection and to provide advice on intervention engagement strategies. The meeting was attended by four professors and members of the NEON study team. During the intervention development stage, there was a single Intervention Design group meeting aimed at discussing particular features of the intervention and how they could be improved. This meeting was attended by two LEAP members and a senior research fellow. As before, all meetings had expenses covered.

To ensure that the evolving intervention operated as intended, the web-applications functions were tested by researchers using dummy accounts as well as data collection tools. This included testing of forms used to collect demographics and outcome data, and interactive features included to provide access to recovery narratives. During the analysis, the full intervention testing costs were deemed necessary for the development of the NEON Intervention on the basis that monitoring routine outcomes and usage data, necessary for the NEON trials, is commonplace in clinical practice (45).

Finally, a feasibility study to evaluate a prototype delivery of the NEON Intervention in a population of people with experience of mental health problems was conducted (37). The user feedback from this feasibility evaluation led to improvements in the intervention, for example, updating the color scheme to resemble the UK NHS website less closely. Digital technologies generally test a prototype of their technology on a sample of potential users for their feedback. Therefore, the full cost of the study was deemed relevant to development of NEON.

#### NEON Intervention delivery resource use

Table 6 summarizes resource use and unit cost categories and sources for the components required to deliver the NEON Intervention.

**Table 6:** Resource use and unit cost input parameters for the delivery stage.

**Resource item**	**Staff members and details**	**Quantity consumed (range)**	**Method**	**Cost per unit^a^ (range)**	**Source**
Web hosting	Amazon Web Services Lightsail	Maximum capacity: 2,000 users	Direct observation	£2.40 per day^b^	[Invoice to the NEON study]
Personnel training:	APM2	2 days	Direct observation	£18.25 per hr (£15.61–£21.80)	[The University of Nottingham's Aug 2021 Clinical Salary Scales]
Administrator	APM3	2 days		£23.90 per hr (£19.92–£28.72)	
	SRF	1 day		£41.34 per hr (£34.47–£49.54)	
Personnel training: Researchers	APM3	2 hrs (1.5–2.5 hrs)	Expert estimation	£23.90 per hr (£19.92–£28.72)	[The University of Nottingham's Aug 2021 Clinical Salary Scales]
	RA	2 hrs (1.5–2.5 hrs)		£28.80 per hr (£23.18–£34.47)	
	SRF	1 hr (0.75–1.25 hrs)		£41.34 per hr (£34.47–£49.54)	
	Researchers	2 days (1.5–2.5 days)		£20 per hr	[Internal communication with NEON study team]
Periodically updating narrative collection	Scaling the cost per narrative	200 narratives per year (100–300 per year)	Expert estimation	£135 per narrative^c^ (£127–£142)	[Authors' calculations]
Web-application maintenance	Technician	20 days per year (20–30 days)	Expert estimation	£300 per day	[Invoice to the NEON study]
Administrative support	APM2	9.8 hrs per week (4.9–14.6 hrs)	Direct observation with assumption	£18.25 per hr (£15.61–£21.80)	[The University of Nottingham's Aug 2021 Clinical Salary Scales]
Advertisement	Paid adverts	N/A	Direct observation	£19.22 per day (duration: NEON Trial) £18.29 per day (duration: NEON-O Trial)	[Invoice to the NEON study]
	APM3	20 days (15–25 days)	Expert estimation	£23.90 per hr (£19.92–£28.72)	[The University of Nottingham's Aug 2021 Clinical Salary Scales]
	RA	18 days (14–22 days)		£28.80 per hr (£23.18–£34.47)	
	SRF	2 days (1–3 days)		£41.34 per hr (£34.47–£49.54)	
Intervention engagement	RA	58 hrs (44–72 hrs)	Expert estimation	£28.80 per hr (£23.18–£34.47)	[The University of Nottingham's Aug 2021 Clinical Salary Scales]
	SRF	20 hrs (15–25 hrs)		£41.34 per hr (£34.47–£49.54)	
Safeguarding	CI	5 hrs (3.75–6.25 hrs)	Expert estimation	£69.91 per hr (£46.49–£102.26)	[Glassdoor March 2022]

APM2, Administrative, Professional and Managerial level 2 (APM2); APM3, Administrative, Professional and Managerial level 3 (APM3); CI, Clinical Investigator; hr, hour; RA, Research Associate; SRF, Senior Research Fellow.

As per the University of Nottingham's “Normal Full-time Working Week,” it was assumed that the number of hours worked per week was 36.25 h for all professionals. Given there were 215 working days in 2021 (including 30 days annual leave, 8 public holidays, and 7 university holidays/closures), the number of hours worked per year was assumed to be 1,559 h.

^a^The unit costs used in the base case model contain direct salary plus on-costs (employer's national insurance contributions, employer's Universities Superannuation Scheme (USS) pension contribution, and the apprenticeship levy).

^b^The web hosting cost per day is derived by dividing the total cost from the invoiced bills (converted $USD to £GBP) by the number of days hosting services were supplied as of the time of this research (1st March 2020 to 31st March 2022). The total cost of web hosting during the 760-day period was £1,823.92 giving a cost per day at £2.40.

^c^The cost to periodically update the narratives was calculated by dividing the total cost of curating the narratives by the number of narratives in the collection at the time of this research, then rescaling to the selected narrative per year amount. The total cost of curating the narratives was £88,640.09 (low: £83,954.30; high: £93,325.89) and the number of narratives currently in the collection was 659 giving a cost per narrative at £135.

The web hosting capacity of the site, together with associated cyber-security features, is supplied by Amazon Web Services Lightsail (https://aws.amazon.com/lightsail/). This service allows websites to host a specific number of users for a publicly advertised price. The current intervention is designed to host 2,000 users. Therefore, a specific level of resource use was predetermined. The invoices from the supplier were made over a monthly billing period in US Dollars (USD); the exchange rates were determined by the credit card issuer.

The cost of personnel training for the administrator, within the host university, is referred to as Administrative, Professional and Managerial level 2 (APM2). The number of days spent training the administrator was recorded for each personnel conducting the training (see Table 5). Training costs would need to be incurred every time there is an administrative staff turnover. Therefore, an assumption was made that a new administrator would need to be trained every 1.5 years. Similarly, the cost of personnel training for the researchers who rated recovery narratives using the INCRESE tool is included as a delivery cost. It was assumed that training costs would need to be incurred every year.

The NEON Intervention required new narratives to be introduced into the collection over time to maintain diversity and relevance to users. Firstly, we consulted with the NEON team to estimate that an additional 200 narratives per year would be needed based on preliminary work looking at the diversity of the current NEON Collection. The cost per narrative was calculated from the current collection size of 659 recovery stories then re-scaled to 200 narratives. Although the cost per narrative approach can provide an estimate of the cost to update the narrative collection, it explicitly assumes a linear relationship between the cost and the narrative collection size. There is uncertainty about whether the cost of updating the narrative collection will increase or decrease for newly sourced narratives. In reality, the process of updating the narratives may become more streamlined and productivity gains can be made in coding the narratives. On the other hand, the cost of updating the narrative collection may be greater if sourcing new narratives becomes more cumbersome, e.g., exhausting the number of existing collections to source narratives.

The web-application requires ongoing maintenance to ensure the NEON Intervention can be delivered as intended for its users. There are challenges in costing for web-application maintenance due to the variability in need for maintenance and the broad definition of what maintenance means in practice. In this case, we define web-application maintenance as any change, modification, or update to the web-application codebase to correct faults, to improve performance, or to update the content on the web-application.

To deliver the NEON Intervention, it was assumed that 9.75 h per week of administrative support by an APM2 is required to conduct operational tasks (e.g., intervention engagement support tasks). The hours per week was estimated by assuming a smaller proportion (50%) of the administrative support observed during the NEON trials (19.5 h per week) would be required in a routine operational setting.

Operational activities that may have influenced the NEON Intervention's effectiveness (i.e., Advertising and Engagement strategies) were also considered to be a delivery cost. Advertisement for the study following agreed advertising principles (46) to recruit eligible participants for both NEON trials. Since the effectiveness and types of adverts may have impacted upon the effectiveness of the intervention it is included as a delivery cost. To ensure users of the NEON Intervention were making use of the intervention, engagement strategies (such as message prompting) were used to encourage use. As these strategies encourage the use of the intervention, they may impact the effectiveness of the intervention and are therefore a part of the delivery cost.

Throughout the delivery of the NEON Intervention, the NEON study was responsible for the wellbeing of those using the NEON Intervention. Safeguarding concerns were dealt with during the trials by the clinical principal investigator.

### Sensitivity analysis plan

To assess the impact of our assumptions and parameter uncertainty, all input parameters were varied to their extreme values in one-way sensitivity analysis. To examine the impact of the structural assumptions, scenario analysis was used. The assumptions that were made during the base-case analysis for both the development and delivery of the NEON Intervention and the sensitivity and scenario analyses are shown in Table 7.

**Table 7:** Sensitivity and scenario analysis plan.

**Resource item**	**Baseline assumption(s)**	**Sensitivity analysis**
**Development stage**		
**Curation of the narrative collection**		
Staff time to source recovery narrative	Midpoint resource use	Max/Min resource use
Staff time to secure ethical approval	Midpoint resource use	Max/Min resource use
LEAP workshops	60% resource use	+(-) 25% resource use
Training researchers	Midpoint resource use	Max/Min resource use
**Web-application development**		
Database specification	Midpoint resource use	Max/Min resource use
Recommender system	30% of budget	+(-) 25% resource use
Interaction design	Midpoint resource use	Max/Min resource use
**Additional communication**		
Task meetings	33% resource use	+(-) 25% resource use
International advisory board	Resource use estimate	+(-) 25% resource use
**Testing the intervention**		
Intervention testing	Midpoint resource use	Max/Min resource use
Feasibility study	Resource use estimate	+(-) 25% resource use
**Delivery stage**		
Personnel training (administrator)	Turnover period every 1.5 years	0.5-2.5 years
Personnel training (researcher)	Turnover period every 1 year	0.5-1.5 years
Periodically updating the narrative collection	200 narratives per year	100-300 narratives per year
Web-application maintenance	Midpoint resource use	Max and min resource use
Administrative support (APM2)	50% resource use	+(-) 25% resource use
Advertising	Midpoint resource use	Max/Min resource use
Intervention engagement	Midpoint resource use	Max/Min resource use
Safeguarding	Midpoint resource use	Max/Min resource use
**Scenario analysis**		
Resource use	Midpoint resource use	Max and min resource use
Wage per hour	Mid-spline salary	Max and min spline salary
Salary	Direct salary plus on-costs*	Direct salary only and direct salary plus on-costs* and overheads
Hours worked per week	36.25	31.25–41.25
Staff time to code the narrative	2-h breaks	0–4-h breaks
Impact of the number of users	No. of users in the intervention arms of NEON and NEON-O Trials	500, 1,000, 2,000 users
Best/worst case	Baseline assumptions	Optimistic/pessimistic assumptions

LEAP, Lived Experience Advisory Panel.

^*^Wages contain direct salary plus on-costs [employer's national insurance contributions, employer's Universities Superannuation Scheme (USS) pension contributions, and the apprenticeship levy].

## Results

### Costs of developing the NEON Intervention

In the base-case, the total number of hours to develop the intervention (excluding resource use external to the NEON team) was 2,709 h (45.2 days). The resource items that required the most personnel time were the staff time to code the narrative collection (1,092 h); the staff time to source recovery narratives (562 h); task meetings (341 h); and LEAP workshops (328 h). The members of staff that contributed the most hours include the coding researchers (1,092 h), the research assistants (895 h), and the senior research fellows (394 h). The cost per unit of staff time varied from £18.25 to £69.91 per hour.

A summary of the estimated costs of developing the NEON Intervention is provided in Table 8. The total cost of developing the NEON Intervention was £182,851. The largest cost components include software development (27%); LEAP workshops (23%); coding the narratives using the INCRESE tool (9%); and researchers' time to source narratives (9%). The total cost of curating the narrative collection was £82,710. The majority of this cost is attributed to the LEAP meetings (50%), coding the narratives using the INCRESE tool (20%), and the researchers' time to source narratives (19%). The total cost of developing the web-application was £64,067. The largest contributions to the development cost of the web-application were the software development (77%); developing the recommender system (12%); and the graphic design (7%).

**Table 8:** Cost of developing the NEON Intervention (base-case).

**Resource item**	Costs (£, 2020/21)*
**Curation of the NEON Collection**	
Staff time to source recovery narrative	15,740
Staff time to secure ethical approval	5,013
Collection organizers	1,650
LEAP workshops	41,372
CSG meetings including preparation for meetings	2,555
Training researchers	470
Coding narratives using INCRESE	21,840
**Web-application development**	
Database specification	1,349
Software development	49,279
Recommender system and integrating into the codebase	7,531
Graphic design	4,560
Interaction design	1,349
**Additional communication**	
Task meetings	12,575
International Advisory Board meetings	670
Intervention Design Group meetings	131
**Testing the intervention**	
Intervention testing	8,139
Feasibility study	8,629
**Total cost**	**182,851**

CSG, Collection Steering Group; INCRESE, inventory of characteristics of recovery stories; LEAP, Lived Experience Advisory Panel.

^*^Costs have been rounded.

### Costs of delivering the NEON Intervention

In the base-case, the total number of personnel hours to deliver the intervention during the NEON trial was 1,708 h (28.5 days). In the NEON-O trial, the total number of personnel hours to deliver the intervention was 1,776 h (29.6 days). The resource items that require the most personnel time were the administrative support (54%); maintenance (19%); advertising (17%). The cost per unit of resource use varied between £2.40 and £125.

A summary of NEON Intervention delivery costs is provided in Table 9. The total cost of delivering the NEON Intervention during the NEON trial was £118,663 (£321 per user). In the NEON-O trial, the total delivery cost of the NEON Intervention was £123,444 (£241 per user). Therefore, the total delivery cost during the NEON trial was 4% lower than during the NEON-O trial. However, the cost per user during the NEON trial was 33% higher than the NEON-O trial. The proportion of fixed costs (advertising, engagement, and safeguarding) was 22% during the NEON trial compared to 21% during the NEON-O trial. The largest cost components include updating the narrative collection (50%); advertising (19%); administration (14%); and software maintenance (11%). The cost of delivering the intervention for a 1-year period is £68,521.

**Table 9:** Cost of delivering the NEON Intervention (base-case).

**Resource item**	NEON (£, 2020/21)*	NEON-O (£, 2020/21)*	Yearly (£, 2020/21)*
Web hosting	1,907	2,005	875
Personnel training (administrator)	1,515	1,593	696
Personnel training (researchers)	951	1,000	437
Periodic updates to the narrative collection	58,593	61,615	26,901
Web-application maintenance	13,068	13,742	6,000
Administrative support	16,669	17,529	7,653
Advertisement	23,111	23,111	23,111
Intervention engagement	2,497	2,497	2,497
Safeguarding	350	350	350
Total cost*	**118,663**	**123,444**	**68,521**

^*^Costs have been rounded.

### Sensitivity analysis

**Figure 1:**
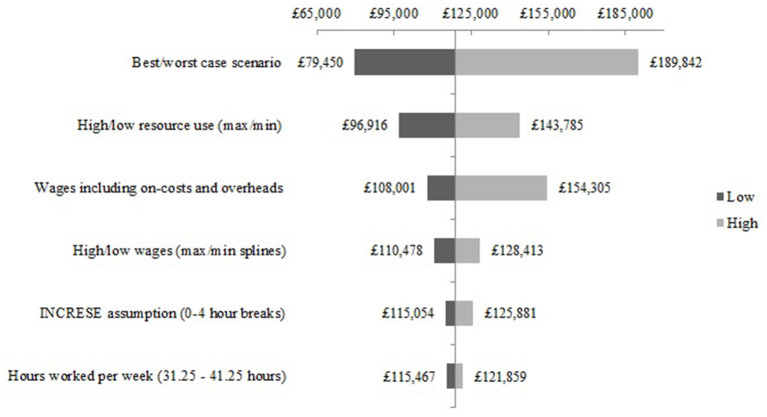
One-way sensitivity analysis - NEON Intervention development costs.

The one-way sensitivity analysis of the most sensitive cost components for NEON Intervention development are presented as a Tornado diagram in Figure 1. Uncertainty in the cost of the LEAP meetings had the greatest impact on the base-case estimate. Specifically, the total cost may be 5.8% higher or lower than the base-case estimate. The difference between the extreme values of the LEAP meetings, task meetings and recommender system development are £21,041; £17,910; and £11,513, respectively. Varying cost components such as intervention testing and efforts to secure ethical approval had a relatively smaller effect on the total development cost. By varying these costs to their extreme values, the impact on total cost of intervention testing and effort to secure ethical approval was +/- £1,296 and +/-£1,003, respectively. Other components had comparatively little effect on the overall development cost such as the database specification (+/- £150).

**Figure 2:**
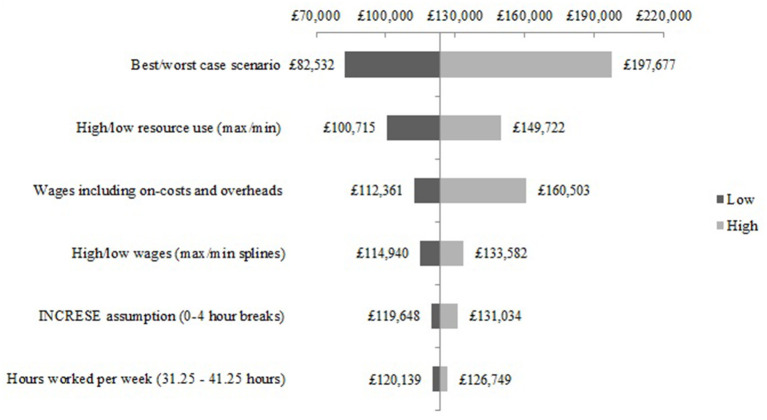
One-way sensitivity analysis - NEON Intervention delivery costs during the NEON trial.

**Figure 3:**
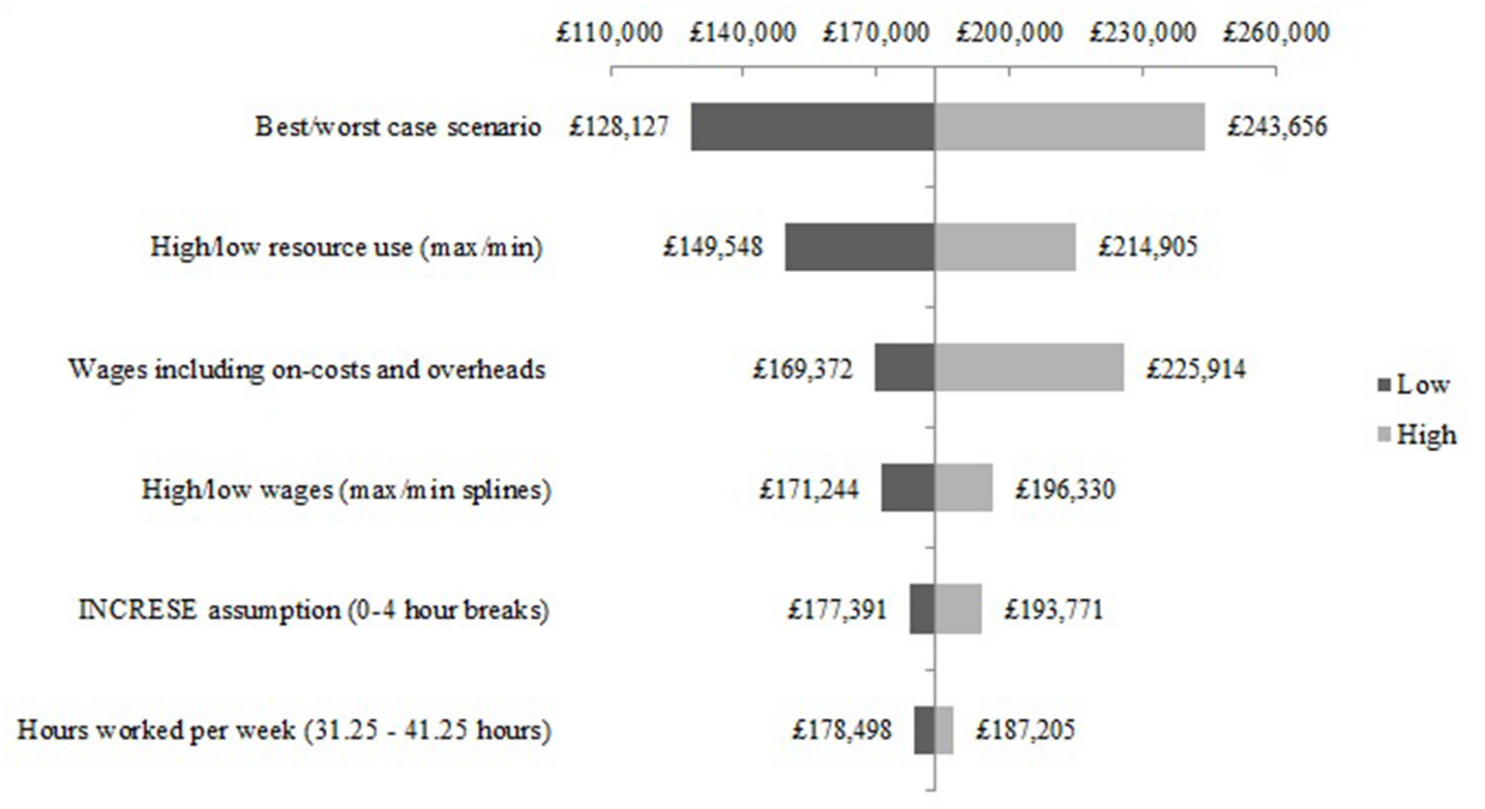
One-way sensitivity analysis - NEON Intervention delivery costs during the NEON-O trial.

The one-way sensitivity analysis of the cost components for the NEON Intervention delivery during the NEON trial are presented as a Tornado diagram in Figure 2. Uncertainty in the cost of administration was shown to have the largest effect on the base-case estimate. Specifically, the total cost may be 7% higher or lower than the base-case estimate. Cost components such as the web-application maintenance and updating the narrative collection had a relatively smaller effect on delivery costs. For example, the impact on the total delivery cost of varying web-application maintenance to its extreme values was £6,534 higher or lower than the base case estimate. Cost components such as engagement had a relatively small effect on the delivery of the NEON Intervention during the NEON trial. The impact on the total delivery cost of varying the engagement component to its extreme values was 0.5% higher or lower than the base case estimate. Similar results can be seen in the one-way sensitivity analysis of the cost components for the NEON Intervention delivery during the NEON-O trial (see Figure 3).

**Figure 4:**
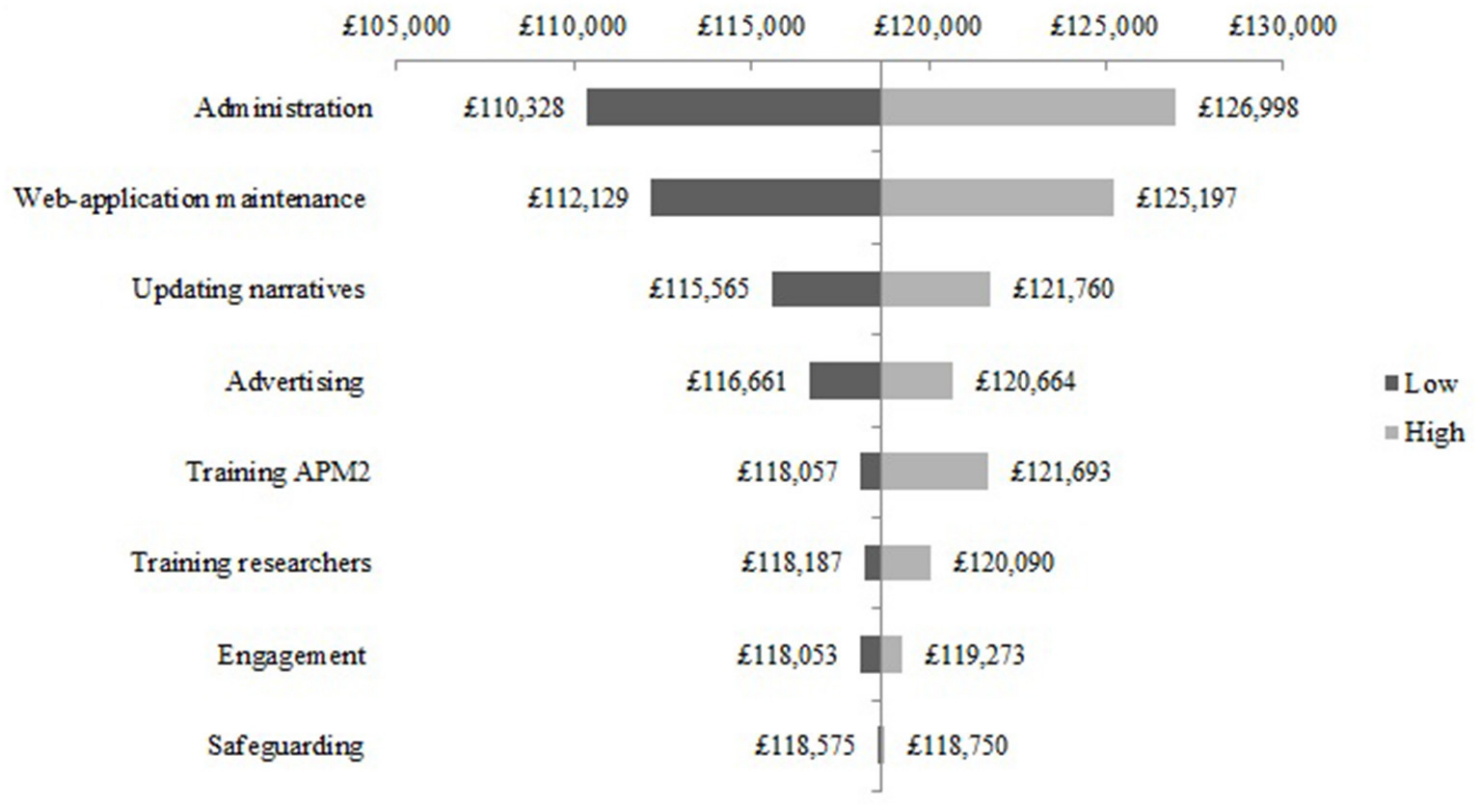
Scenario analysis - NEON Intervention development costs.

The scenario analysis of the structural assumptions for developing the NEON Intervention are presented as a Tornado diagram in Figure 4. The impact on the total cost of the best and worst case scenario analyses show a feasible total cost range to develop the NEON Intervention. The cost of developing the NEON Intervention given the best possible scenario is £54,724 lower than the base-case estimate of £181,851. Similarly, given the worst possible scenario, the cost of developing the NEON Intervention is £60,805 higher than the base-case estimate.

**Figure 5:**
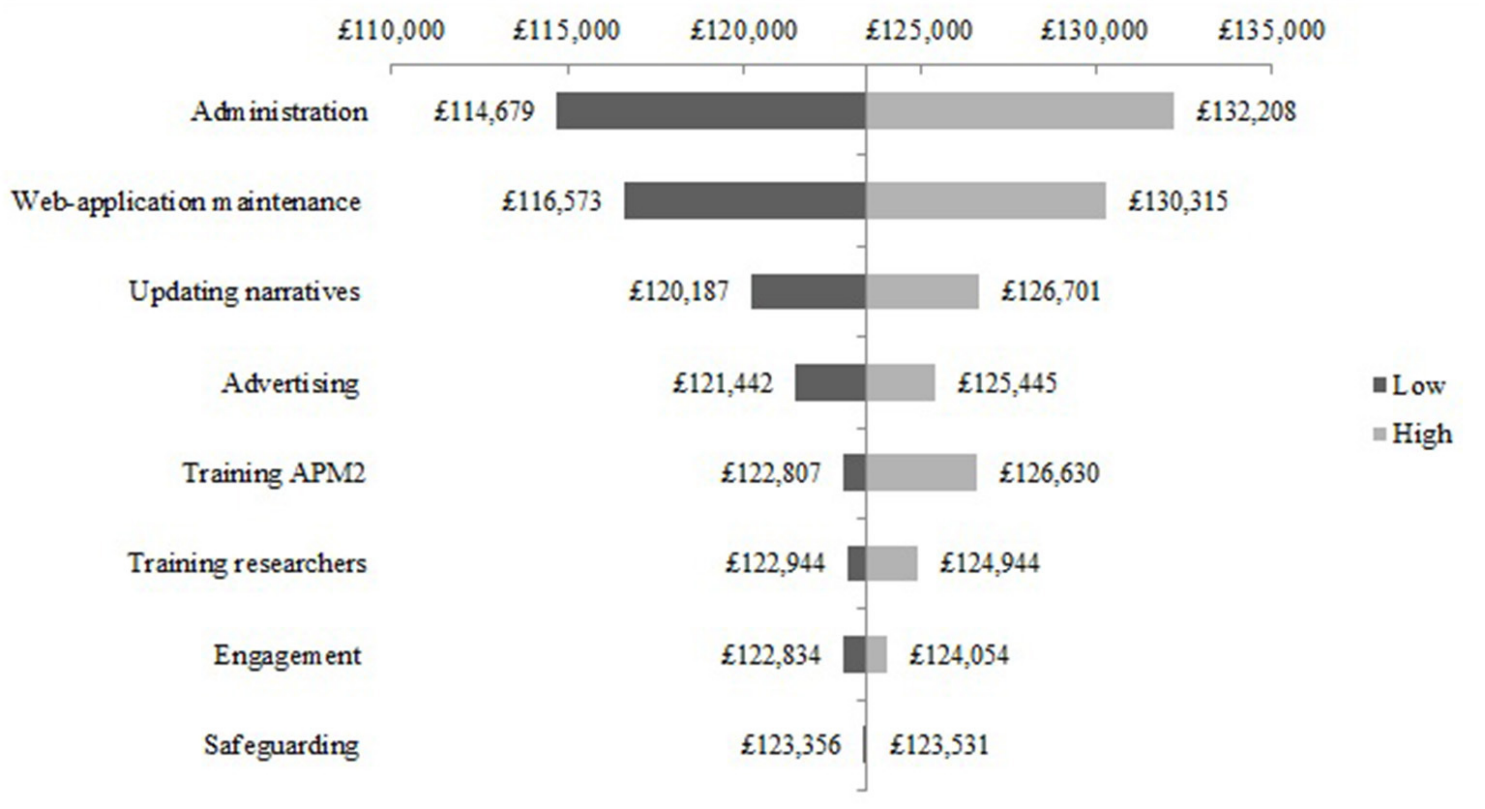
Scenario analysis - NEON Intervention delivery costs during the NEON trial.

**Figure 6:**
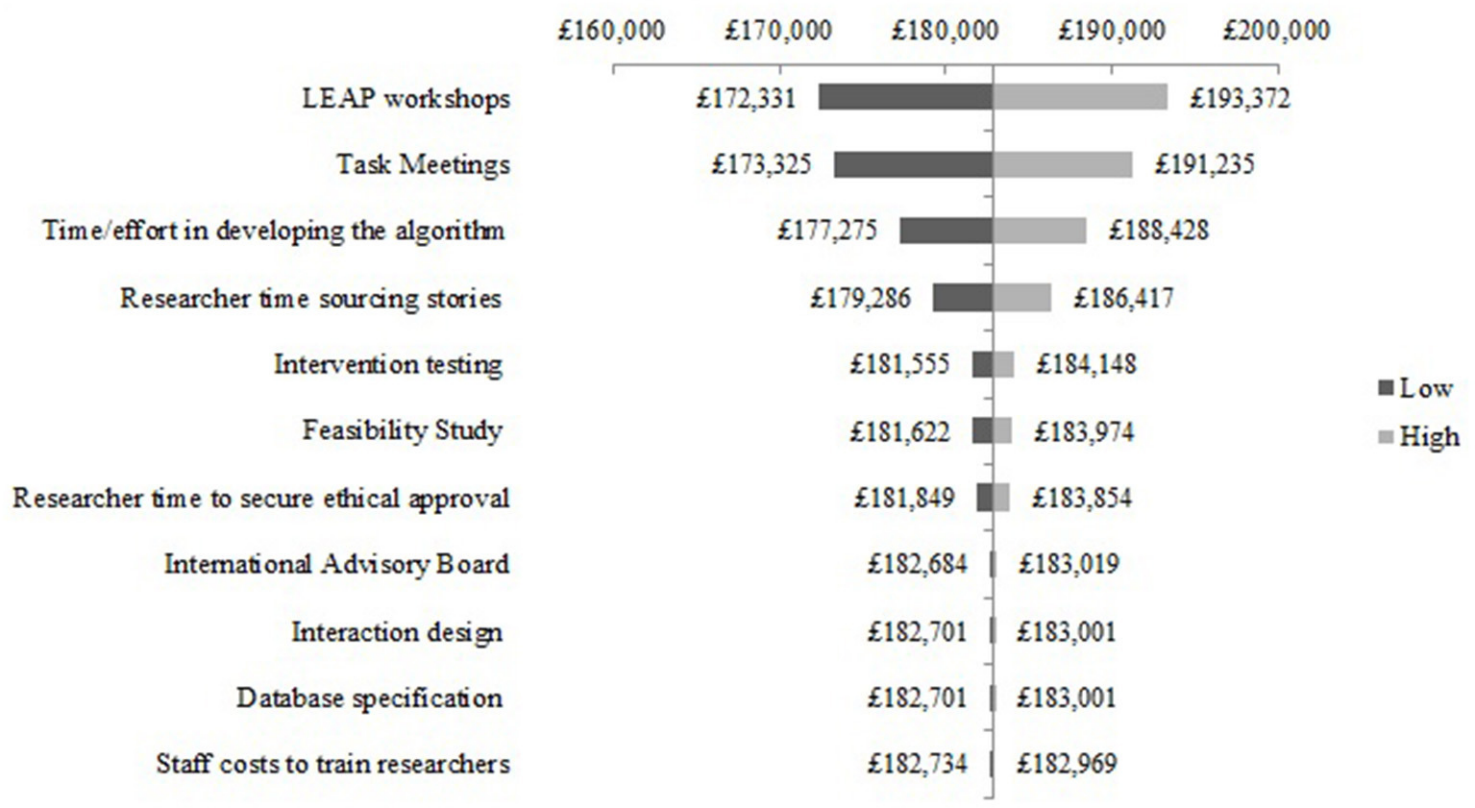
Scenario analysis - NEON Intervention delivery costs during the NEON-O trial.

The scenario analysis of the structural assumptions for the NEON Intervention delivery during the NEON trial are presented in Figure 5. The best-case scenario is £39,213 lower than the base-case estimate, and the worst-case scenario cost is £71,180 higher than the base-case estimate. Similar results can be seen in the scenario analysis of the structural assumptions for the NEON Intervention delivery during the NEON-O trial (see Figure 6).

As expected, increasing the number of users, reduced the cost per user year, such that 500, 1,000, or 2,000 users cost £137.04, £68.52, £34.26, respectively.

## Discussion

### Key findings

We identified several resource use categories for intervention development and delivery through a literature review and work with experts on the research team. Developing the NEON Intervention cost £182,851, which was largely attributed to building the NEON Collection (£82,710) and developing the web-application (£64,067). The curation costs were mostly made up of LEAP meetings (50%); narrative coding using the INCRESE tool (20%); and the researchers' time to source recovery narratives (19%). The largest components of the web-application costs included software development (77%); developing the recommender system (12%); and the graphic design costs (7%).

Delivering the NEON Intervention during the NEON trial and NEON-O trial costed £118,663 and £123,444, respectively. This equates to £349 (NEON trial) and £241 (NEON-O trial) per user. Over an annual period, the NEON Intervention cost was £68,521. Delivery costs were driven by updating the narrative collection (50%); advertisement (19%); and administrative support. Similarly to other studies (20), we found that total costs are dependent on usage rates, which are difficult to predict. Due to a high proportion of fixed costs, costs per user were high for the lower caseload in NEON-O but would be expected to decline with increasing volume of use.

### Strengths and limitations

The strength of our study is the use of published guidelines where available to inform our costing methods. We have provided detailed and transparent reporting of cost components and we have utilized multi-disciplinary input into identifying categories, including strong service user involvement. We have carried out sensitivity analysis to make explicit which parameters are associated with uncertainty, and the direction and magnitude of that uncertainty.

The limitations of our study include the necessary use of expert opinion sources where primary data were not collected or available. Due to limitations in patient-level resource use data availability, we have had to use top-down costing methods rather than bottom-up, or micro-costing approaches, which arguably does not provide a patient-level cost sensitive to different levels of usage by individual patients. This limitation is common to costing DHIs, due to the nature of the costs involved (47).

Defining a starting point for development costs necessarily meant excluding early development costs. As with other research-led endeavors, this work is supported by the accumulation of an existing knowledge base and the experience and expertise of the research team, as well as the opportunity cost of the researchers. This can be conceptualized using the health service research payback model which examines the complex interaction and costs of early and late publicly-funded research, and their effect on research outputs (48). There has been a substantial program of earlier research to support the development and testing of the NEON Intervention, which is outside the scope of this work to quantify. Other costs which were outside the scope include the intellectual work involved in the development of the NEON proposal for funding, the other types of resource such as existing collaboration networks which could be accessed for advice, and work conducted in other studies in the research group which may have cross-fertilized the NEON work. We also did not include the RCT costs in our development costs as we explicitly examined development costs up to the point of the beginning of the RCTs. The impact that the trial's findings will have upon the NEON Intervention is currently uncertain. As such, it is unclear what changes the NEON trials may inform to improve, scale, or discontinue the intervention in its current form. Therefore, trial costs were not considered as a development cost during the current development period.

The delivery costs provided here are derived from the delivery of the NEON Intervention during the trials, so it is likely that delivery costs in practice will be different, and health care payers need to be prepared for costs to vary once the intervention is implemented outside a clinical trial environment. How the NEON Intervention is implemented in the NHS will likely determine what resources will be needed in future versions of the intervention. As the NEON trials do not compare the NEON Intervention with a face-to-face version of the same intervention, we are not able to examine the differences in costs (or effectiveness) between these two scenarios.

Assumptions had to be made around tasks and roles taken up by researchers in the trial that would actually be covered by healthcare professionals, administrators, and other members of the healthcare team when the intervention is delivered in practice. Necessarily there are likely to be some differences between trial delivery and practice delivery. For example, the clinical principal investigator coordinated all safeguarding activities during NEON Intervention delivery as part of the RCT. If the NEON Intervention is rolled out in practice, at a substantial scale, then the safeguarding approaches and infrastructure needed will necessarily be different. Safeguarding approaches will need to scale with safeguarding demands, and to take into account different regulatory requirements in everyday practice compared to clinical trials. We have treated advertising costs in the trials as a proxy for accessing patients in practice. Recruitment for the NEON Trial was targeted carefully, for example through the production of several 100 online messages with content specific to a psychosis trial and the design and dissemination of targeted adverts (for example displayed as banners on websites used by health and social care professionals and potential participants). This involved human effort and expertise to design these materials. If the NEON Intervention is rolled out to a general mental health population on a larger scale through online mechanisms, then less targeting of recruitment material might be needed, and individual messages or adverts can also be reused and redeployed. This means that the human cost of generating recruitment material might be less and the dominant cost might be spent on the services that social media companies provide to promote messages to a relevant audience. Especially at the point where the NEON Intervention is deployed on a wider scale, the cost of recruiting one person through social media promotion should be routine to estimate.

Implementation of the NEON Intervention beyond the trials will lie on a spectrum. On the one end, the implementation through statutory mental health services (costs associated with staff awareness and training, implementing safeguarding procedures etc.), through to the implementation *via* primary care or the voluntary sector, to “direct to consumer” (costs associated with advertising), and the value for money offered by these different implementation routes (which may be separate or additive) that could be evaluated in relation to reach and engagement. The most efficient way to implement DHIs like the NEON Intervention is very relevant in a resource-constrained system as implementation methods cost money and affect the effectiveness and cost-effectiveness of the DHI (49).

Other limitations in the delivery costs include the lack of regulatory or scale-up costs, such that these costs may be an underestimate of true costs once the NEON Intervention is implemented in practice. The derivation of costs per user from top-down costs necessarily means that the cost per user is related to the number of users assumed in the calculations. The number of users could be the number of people in each trial, the number of people eligible to use the NEON Intervention in the real world (21), or we would suggest, the maximum capacity of the current technology implementation to provide an appropriate level of quality of service (50) to its users (for example with sufficient responsiveness of interactive features to allow for a satisfying engagement). If we use the number of people in each trial to estimate cost per user, the mean cost per user could be overestimated as the maximum technological capacity consistent with acceptable service quality has not been reached. However, using the number of people eligible to use the NEON Intervention in the real world is not straightforward. This is for a number of reasons: the computational complexity of the algorithms used in the technology may not scale linearly with the size of the user base (meaning that needed server performance and hence server cost may not scale linearly), and at discrete points, technology re-engineering may be needed to maintain service quality, for example by replacing propriety content delivery system with commercially-provided Content Delivery Networks (51). This reflects the development cycle for web-based DHIs. It is similar to most start-ups that proceed through a series of versions of their systems, re-engineering them each time for a larger number of users. Costs associated with capacity-related engineering and enhanced server capacity should be spread across the anticipated number of users, and allocated equally per user. For example, additional costs required to scale the intervention from 2,000 to 5,000 users should be spread equally across those 3,000 extra users to prevent spikes in costs per user. In the final report for the NEON trials, we will examine this issue further and look at scenarios for future costs for the NEON Intervention as it is scaled up to a range of anticipated user base sizes.

Given the evolving nature of DHIs, input parameters into economic evaluations (like delivery costs) need to be re-examined at different stages of the intervention capacity. It is likely that certain cost components will change over time. Hosting costs are likely to reduce due to increases in process performance and reductions in storage cost per unit (Moore's Law) (52), and salaries may increase (given human resources have quite a large impact on the delivery, this is important).

Another issue beyond the scope of this study is the likelihood that the intervention will be delivered differently once it is implemented in practice. This will have an impact not only on resource use but also on effectiveness of the intervention. However, the data we have provided will provide healthcare providers with approximations of the resources required to deliver the intervention in practice.

### Comparisons with existing evidence

We are not aware of a DHI that has focused on the use of narratives to support recovery in mental health, so it is not possible to compare our development and delivery costs with another equivalent intervention or directly relevant costing study. It is also unclear as to the utility of comparing the costs we have estimated with other mental health DHIs costs, due to the lack of comparability of costing methods between studies. The reporting of different development and delivery cost components of mental health DHIs varies, making meaningful comparison difficult (11, 15).

Development costs are reported in a minority of economic evaluations, as they are usually seen as sunk costs and not relevant to the health and social care provider. The list price of commercially developed healthcare products such as pharmaceuticals are perceived to include development costs and thus recoup those costs. Development costs are more difficult to determine for DHIs. Opinions differ on the inclusion of development costs, and it is the perspective that should determine whether they are included or not (47). When development costs are included, a judgement has to be made regarding when development costs are considered to begin and end, as we had to make in our study, and this judgment can have a significant effect on the overall costs derived.

When reported, development costs vary from as little as £19,000 (53) to £500,000 (54), but whether this variation stems from the varied nature of the DHIs or from the methods used to collect resource use data is challenging to untangle. However, they can influence whether development occurs, so explicit methods for deciding on resource categories to include, methods of resource use data collection and sources of unit costs should be explicitly given in any study. We used a similar approach to Joshi and colleagues who reported explicit methods to derive development cost for a digital program for training community health workers to deliver treatment for depression in rural India (22). Similarly to our study, they reported that staffing costs constituted 61% of development costs.

In a recent review of methods used in economic evaluations of mental health DHIs, 16 out of 66 did not report staffing costs as part of delivery costs (15). Given the human resource intensive nature of DHI delivery as found in our study, this omission would lead to a significant underestimate of costs. Only 14 out of 66 studies included costs for website maintenance and hosting, again we found that this constituted a significant delivery cost.

We used the NICE guidance to support our costing methodology. However, there have been questions raised surrounding how fit for purpose this guidance is for costing DHIs in practice (21). Like other economic evaluation guidelines, although they provide a costing framework, there is little practical guidance on specific costing (47). We identified several resource use categories in intervention development and delivery, and future developers of DHIs should consider including these categories, and working with relevant experts to identify any further categories specific to their intervention. We also recommend keeping formal resource use records to allow easier derivation of costs. Since DHIs (in keeping with commercial web-applications) are typically subject to ongoing periods of maintenance punctuated by periodic redevelopment and reengineering work which will have an impact on the cost per user, we would advocate for the collection of a broad range of case studies providing evidence on the cost over the life of a DHI of ongoing development work. We have excluded conceptual work conducted before the NEON program was funded, and in the early stages of the NEON program. Future studies might consider approaches to costing in such activities.

Human resources constituted a large proportion of our development and delivery costs. These estimates were affected by uncertainties around which costs to include, other than salary, so we followed the PSSRU approach to include employers' costs, as well as estates and indirect costs for the organization employing that person (55). Our sensitivity analysis demonstrated that inclusion of these latter costs had a significant effect on overall costs.

## Conclusion

This study makes two knowledge contributions. First, it provides a usable estimate of the cost of developing and implementing a DHI from software and intervention development, through feasibility testing and up to commencement of the definitive trial. This can be used to inform commissioning of new DHIs in general, giving explicit consideration to all the different types of resources required, and the quantity and cost, as well as specifically, the implementation of the NEON Intervention. Second, the costing challenges which have been identified indicate the need for updated best practice guidance for economic evaluation of DHIs by NICE and other clinical and funding agencies.

## Relevance for clinical practice

The NEON Intervention is intended for widespread use as a low-cost self-management intervention. Two uses are envisaged: adjunctive to clinical treatment and direct access. This study identifies the costs associated with population-level roll-out of the NEON Intervention. In relation to use within services, the staff costs needed to support access are identified. In relation to direct access, the public health costs associated with maintaining and developing the intervention are estimated. We are currently evaluating the effectiveness of the NEON intervention based on the two RCTs described in this study. Data analysis is near completion and will be published separately building on the findings in this study. These findings will inform decision-making about whether, and how, to implement the NEON Intervention at scale.

## Data availability statement

The original contributions presented in the study are included in the article/Supplementary material, further inquiries can be directed to the corresponding author.

## Ethics statement

The studies involving human participants were reviewed and approved by Leicester Central Research Ethics Committee, 19/EM/0326. Informed consent to participate was obtained from all participants using an online form. The patients/participants provided their written informed consent to participate in this study.

## Author contributions

RE, SG, LP, and SR-E contributed to the conception and design of the study. LP carried out the data collection and analysis and wrote the first draft of the manuscript. SR-E, SG, and RE wrote sections of the manuscript. JL-B, FN, CB, AG, JN, DQ, and SB contributed to the data collection. JL-B and FN provided feedback on the analysis. MS is the chief investigator for this trial. RE is the lead health economist. All authors have read, commented, and reviewed the manuscript.

## Funding

This study was funded by the NIHR [Personal experience as a recovery resource in psychosis: Narrative Experiences Online (NEON) Programme (RP-PG-0615-20016)]. The funder was not involved in the development of the methods, data collection, analysis or writing of this manuscript.

## Conflict of interest

The authors declare that the research was conducted in the absence of any commercial or financial relationships that could be construed as a potential conflict of interest.

## Publisher's note

All claims expressed in this article are solely those of the authors and do not necessarily represent those of their affiliated organizations, or those of the publisher, the editors and the reviewers. Any product that may be evaluated in this article, or claim that may be made by its manufacturer, is not guaranteed or endorsed by the publisher.

## Author disclaimer

The views expressed are those of the author(s) and not necessarily those of the NIHR or the Department of Health and Social Care.
